# Unusual manifestations of acute Q fever: autoimmune hemolytic anemia and tubulointerstitial nephritis

**DOI:** 10.1186/1476-0711-11-14

**Published:** 2012-05-18

**Authors:** Serdal Korkmaz, Nazif Elaldi, Mansur Kayatas, Mehmet Sencan, Esin Yildiz

**Affiliations:** 1 Department of Internal Medicine, Faculty of Medicine, Cumhuriyet University, Sivas, Turkey; 2 Department of Infectious Diseases and Clinical Bacteriology, Faculty of Medicine, Cumhuriyet University, Sivas, Turkey; 3 Department of Pathology, Faculty of Medicine, Cumhuriyet University, Sivas, Turkey; 4 Department of Internal Medicine, Cumhuriyet University, Faculty of Medicine, 58140, Sivas, Turkey

**Keywords:** Q fever, Pneumonia, Autoimmune hemolytic anemia, Tubulointerstitial nephritis

## Abstract

Q fever is a worldwide zoonotic infection that caused by *Coxiella burnetii,* a strict intracellular bacterium. It may be manifested by some of the autoimmune events and is classified into acute and chronic forms. The most frequent clinical manifestation of acute form is a self-limited febrile illness which is associated with severe headache, muscle ache, arthralgia and cough. Meningoencephalitis, thyroiditis, pericarditis, myocarditis, mesenteric lymphadenopathy, hemolytic anemia, and nephritis are rare manifestations. Here we present a case of acute Q fever together with Coombs’ positive autoimmune hemolytic anemia (AIHA) and tubulointerstitial nephritis treated with chlarithromycin, steroids and hemodialysis. Clinicians should be aware of such rare manifestations of the disease.

## Background

Q fever is a worldwide zoonosis caused by *Coxiella burnetii*, a strict intracellular bacterium. Human infection results mainly from infected aerosols generated by farm animals when they give birth or abort. The disease is classified into acute and chronic forms. Chronic form is generally associated with endocarditis, osteomyelitis, infected vascular aneurysms, or infected intravascular prostheses [1]. Acute Q fever often presents either as a self-limiting flu-like illness, hepatitis, or pneumonia. The most frequent clinical manifestation of acute form is a self-limited febrile illness which is associated with severe headache, muscle ache, arthralgia and cough [2]. Other rare manifestations are seizure and coma, meningoencephalitis, thyroiditis, pericarditis, myocarditis, mesenteric lymphadenopathy, pancreatitis, hemophagocytosis, hemolytic anemia, transient hypoplastic anemia, and epididymoorchitis [3]. Here we present a case of acute Q fever together with Coombs’ positive AIHA and tubulointerstitial nephritis treated with chlarithromycin, steroids and hemodialysis.

## Case presentation

A previously healthy 39-year-old man who was an officer in a city hospital presented with a 10-day history of fever, chills, fatigue, sweats, and muscle aches. He noted no complaints of cough and sputum but dyspnea and jaundice which began three days ago before presentation and gradually worsening over 24–48 hrs. He had no history of exposure to an animal and travel to rural areas. His temperature was 39^0^C (102.2^0^F), his pulse was 96 beats/min, his respiration rate was 24 breaths/min, his systolic blood pressure was 110 mmHg, and his oxygen saturation was 96% on room air. The patient seemed icteric and lung auscultation showed bibasilar crackling. Findings of additional examination were unremarkable. Arterial blood gas analysis revealed a marked respiratory alkalosis (elevated pH and marked decline in pCO_2_) and mild hypoxemia (pO_2,_ 75 mm Hg).

**Figure 1:**
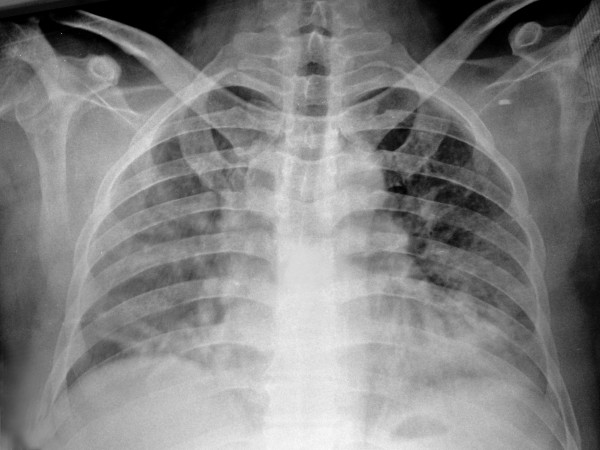
**a) Multiple pulmonary infiltrates. b)** Widespread patchy ground-glass opacities throughout both lungs and bilateral pleural effusion.

The hemoglobin concentration was 4.4 g/dL, WBC count was 24 X 10^3^cells/μL and platelet count was normal. Blood film examination showed 90% of neutrophils with toxic granulations and erythrocyte morphology with hypochromia, anisocytosis and spherocytosis. The levels of blood urea nitrogen (BUN), creatinine, electrolytes, vitamin B12, folate and aspartate aminotransferase (AST) and alanine aminotransferase (ALT) were normal. The serum lactate dehydrogenase (LDH) level was 414 U/L (normal range, 125–240 U/L) and total bilirubin was 5.8 mg/dL (normal range, 0.3-1.2 mg/dL) with a rate of 3.0 mg/dL indirect bilirubin. The corrected reticulocyte count was 4% (normal range, 0.5-2.5%). A routine urinalysis was normal and the fecal occult blood test was negative. The erythrocyte sedimentation rate (ESR) was 100 mm/h, and the C-reactive protein (CRP) level was 272 mg/L (normal value, <8 mg/L). Chest radiography showed multiple pulmonary infiltrates and an additional CT of thorax widespread patchy ground-glass opacities throughout both lungs suggesting acute interstitial or viral pneumonia and bilateral pleural effusion (Figure 1a and Figure1b). The direct Coombs’ test (antiglobulin test) for explaining of hemolysis was positive. The patient was admitted to the intensive care room, and he began receiving intravenous fluids, empirical ampicillin/sulbactam intravenously plus oral clarithromycin for clinical diagnosis of community acquired pneumonia, and methylprednisolon intravenously (1 mg/Kg daily) for controlling hemolytic process.

A total of 4 unites of erythrocyte suspension were administered in 24 hours and the hemoglobin concentration increased to 8.0 g/dL. Despite the clinical situation of him was good, the patient's laboratory values worsened on the second day of admittance. The BUN rose to the level of 43 mg/dL (normal range, 5–25 mg/dL) and the creatinine rose to the level of 3.4 mg/dL (normal range, 0.7-1.2 mg/dL). The AST level was 1388 U/L (normal range, 5–40 U/L) and the ALT level was 832 U/L (normal range, 5–54 mg/dL). Total bilirubin level increased to 9.4 mg/dL (indirect content, 4.1 mg/dL), and the LDH level to 4822 IU/L. The total creatine kinase level was 1515 U/L (normal range, 38–176 U/L). Performed sequential electrocardiograms and echocardiography test showed no cardiac pathology. Thyroid function tests were within normal values. A serum sample for diagnosing of Hantavirus infection, Crimean-Congo hemorrhagic fever virus (CCHFV) infection, and acute Q fever and a throat swab specimen for diagnosing of H_1_N_1_ influenza were sent to the Refik Saydam Hifzissihha Institute, Ankara, Turkey. Serological markers indicating acute infection with Epstein-Barr virus, cytomegalovirus, herpes simplex virus, parvovirus, varicella zoster virus, mumps virus, measles virus, and rubella virus were all negative. The detailed viral hepatitis markers andBrucella-specific Wright tube agglutination test were also negative. The blood and urine cultures for bacteria yielded no growth.

**Figure 2:**
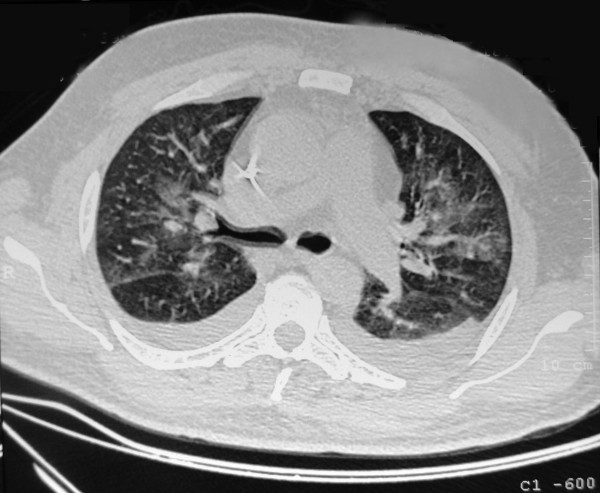
Histologic findings in the kidney demonstrating focal tubular atrophy, interstitial edema and infiltration with lymphocytes and eosinophils, and hyalen cylendirs (hematoxylin and eosin stain; original magnification, X20).

His blood hemoglobin concentration decreased again to 5 gr/dL on the 3^rd^ day and cold agglutinin test was found to be negative on the same day. The blood creatinine rose to the level of 8.4 mg/dL on the day 4^th^, and anuria developed. After evaluation, it was decided to take the patient in hemodialysis treatment 3 times a week. A percutaneous renal biopsy on the day 12^th^ demonstrated acute tubulointerstitial nephritis (TIN, Figure 2). Serologic and virologic analyses with ELISA and real time-PCR method for Hantavirus infection, CCHFV and PCR method for H_1_N_1_ influenza were negative. Serum sample was found to be positive against *C. burnetii* by immunofluorescence assay (IFA) and indicated an acute Q fever infection with the titer of 1:512 for IgG, 1:64 for IgM against phase II and a negative serology against phase I antigen. The patient continued receiving antibiotics and hemodialysis treatment for 14 days. He became afebrile after 5 days of antibiotic and steroid treatment, his clinical condition improved in 10 days, AST and ALT returned to normal levels within 15 days. The blood creatinine returned to normal levels 16 days after hemodialysis started and then hemodialysis treatment no longer needed. A total of 18 units of erythrocyte suspension were administered during the hospitalization period and the blood hemoglobin concentration was 10.9 g/dL on the 16^th^ day of admission. Total bilirubin returned to normal levels after 21 days of admission. The pulmonary infiltrates improved after 21 days of antibiotic therapy. Steroid therapy was continued for 3 months and discontinued by reducing the dose over time. The patient improved without any complications and he was uneventful after 6 months of follow up.

## Discussion

Pneumonia and hepatitis are frequently seen in acute Q fever and there are two additional manifestations in our patient: TIN with acute renal dysfunction and Coombs’ positive AIHA. Glomerulonephritis, attributed to immune-complex deposition and a well-recognized feature of chronic form of Q fever, is described less frequently in acute form [4]. Since a small number of cases have reviewed systematically, the incidence of renal complication in Q fever is unknown. In a recent retrospective study performed in 54 acute Q fever patients have demonstrated 33% of patients having renal failure [5]. Acute TIN is an important cause of acute renal failure, and often associated with use of drugs and various infections. Those most commonly implicated are antibiotics (penicillins, cephalosporins, sulfonamides and rifampicin) and non-steroidal anti-inflammatory drugs although cases of TIN have been linked to many medications [6]. Clarithromycin has also been reported previously as an aetiological factor in TIN [7]. Despite using two aetiologic agents (ampicillin and clarithromycin) in the patient for developing TIN, we believe that it has occurred due to Q fever. Firstly, the patient had a history of using any medications before referring to the hospital and he received those antibiotics only for a day before rising blood BUN and creatinine levels. These are unlikely to have contributed to the renal complication. Secondly, extremely lower hemoglobin concentration, a higher bilirubin level and a positive direct Coombs’ test on admittance suggesting strongly autoimmune events. The other mechanism for renal involvement by an infection agent is via direct parenchyma infiltration or a systemic inflammatory response against the bacteria. Rickettsial organisms in the tubular epithelium glomeruli and interstitium of the kidney in a fatal case of Q fever have been demonstrated [8].

Apart from the present case, there are only a few case reports concerning renal complications associated with acute Q fever in the literature in English. A case of acute renal failure in a male patient with acute Q fever who had a febrile illness, pneumonia, and a skin rash was described [9]. The authors reported that there were proteinuria and hematuria in the patient but renal biopsy was not done to confirm suspected glomerulonephritis. They stated that renal failure improved in the patient without dialysis treatment. Korman et al. [10] also reported a male case of acute diffuse proliferative and exudative glomerulonephritis, resolving spontenously, associated with acute Q fever. Antiphospholipid antibodies may also accompanied with acute renal failure in Q fever. Tolosa-Vilella et al. [11] described a case of acute Q fever with mesangioproliferative glomerulonephritis and immunglobulin (Ig) G antibodies to cardiolipin and lupus anticoagulant in acute-phase serum samples. A recent case report has noticed a female patient with postinfectious glomerulonephritis associated with acute form of the disease [12]. Sometimes renal failure in Q fever may not to be related with acute glomerulonephritis or TIN as in our case. A case of acute Q fever associated by extreme rhabdomyolysis and consecutive acute renal failure treated with continuous venovenous hemodiafiltration was presented [13].

Interaction of *C. burnetii* with the host's immune system is complex and still poorly understood. Immune control of *C. burnetii* is T-cell dependent but does not lead to *C. burnetii* eradication. The organism is able to grow and multiply within phagolysosomes [14]. The expression of antigens specific to *C. burnetii* in the membranes of infected host cells [15] supports the hypothesis that infected cells are detected by the immune system and lysed by antibody-dependent cellular cytotoxicity by monocytes and other effector cells. Specific immunoglobulins are secreted following infections. IgG is mainly directed against phase II antigen, whereas IgM is directed against both phase I and II cells [16]. Chronic infections are believed to be a result of immunological reactions and/or defects [14,16]. During chronic Q fever, the immune response is ineffective and may also be harmful, causing leucocytoclastic vasculitis and glomerulonephritis [16]. A variety of autoantibodies have been described in Q fever, including antismooth muscle and antimitochondrial antibodies, ANA, rheumatoid factor, and cold agglutinins [17]. All of these autoantibodies were negative in our case.

Seroconversion to *C. burnetii* develops usually 7–15 days after onset of clinical symptoms, and antibodies against phase I and phase II appear in the blood. The presence of both an anti-phase II IgG titer of ≥1:200 and IgM titer of ≥1:50 is 100% predictive of acute Q fever. However, such results are observed only in 10% of patients during the second week following the onset of symptoms [18]. A clinical and laboratory study which was performed in 22 patients with acute Q fever showed that fifth day serum samples had a sensitivity of 80% in the diagnosis of acute Q fever and sixth to eleventh day serum samples had a sensitivity of 83% by the IFA technique [19]. However, cross-reactivity with other infectious agents such as *ChlamydiaBartonella* and *Legionella* species has been observed especially in chronic Q fever [20-22]. Two hundred and eleven serum samples of acute and chronic Q fever by the IFA test revealed that four serum samples (1.9%) had antibodies against both *C. burnetii* and *Legionella pneumophila*[20]. More than 50% of patients with chronic Q fever had sera containing cross-reacting antibodies to *Bartonella henselae* or *Bartonella quintana*[21]. It has been proposed that a differential diagnosis among the infections is easily established when quantitative antibody titers against both anti-phase I and anti-phase II *C. burnetii* antigens are determined [18]. Because of its protean manifestations, leptospirosis mimics many other infectious diseases including Q fever with pneumonia and it must also be differentiated from Q fever [23].

Various mechanisms are responsible for hemolytic anemia in the course of an infection including direct invasion of red blood cell by the pathogen, toxin production, mechanical effects and immune-mediated hemolysis. Immune mechanisms can also be divided into autoimmune, antigen-antibody complexes and polyagglutination reactions [24]. The patient was anemic on admittance and totally 18 units of erythrocyte suspension were administered during the hospitalization period. The negativity of cold agglutinins, positivity of direct and indirect Coombs’ tests and a higher reticulocyte count suggested strongly that AIHA occurred due to autoimmune events needing steroid therapy. Warm and cold agglutinins can occur during the infectious and non-infectious events and the most of AIHAs are caused by warm antibodies, whereas cold antibodies are less commonly detected. The direct Coombs’ test is the first step in diagnosing of AIHA, and the distinction of warm and cold agglutinins can help in the differential diagnosis of the cause of AIHA. In warm antibody AIHA, standard first line therapy is corticosteroids with or without high dose of immunoglobulin, whereas splenectomy is considered second-line therapy. Patients with cold agglutinins are refractory to steroids and splenectomy [25]. Since the warm and cold agglutinin tests were not available in our institution, these agglutinins could not be identified in the patient for the explaining of the cause of AIHA. Because responded well to the corticosteroid therapy, we think the cause of AIHA is warm agglutinins.

Despite the recommended therapy regimen for Q fever is doxycycline, clarithromycin is a reasonable treatment for acute Q fever [26]. The corticosteroid therapy had two useful effects on our patient. First, it eliminated the autoimmune hemolytic process and therefore, the patient did not need any blood transfusions Second, being useful for inflammatory events, it relieved the renal dysfunction which caused by acute TIN. Additionally, hemodialysis program contributed to control of uremic symptoms in the patient.

## Conclusions

Acute Q fever may be presented as both renal dysfunction and AIHA. Therefore, the clinicians should be aware of such manifestations. Establishment of diagnosis and treatment procedure is simple and is mentioned above. Only it must be done cautiously.

## Consent

Written informed consent was obtained from the patient for publication of this Case report and any accompanying images.

## Competing interests

The authors declare that they have no competing interests.

## Authors’ contributions

SK and NE, ideated this case-report and did most of the writing, supported by MS. MK and EY have been involved in drafting the manuscript. EY has made substantial contributions to acquisition of data. All authors read and approved the final manuscript.
